# Physicochemical and Sensory Attributes of Intact and Restructured Chicken Breast Meat Supplemented with Transglutaminase

**DOI:** 10.3390/ani11092641

**Published:** 2021-09-08

**Authors:** Ana Kaić, Zlatko Janječić, Silvester Žgur, Monika Šikić, Klemen Potočnik

**Affiliations:** 1 Department of Animal Science and Technology, Faculty of Agriculture, University of Zagreb, Svetošimunska Cesta 25, 10002 Zagreb, Croatia; monika.sikich@gmail.com; 2 Department of Animal Nutrition, Faculty of Agriculture, University of Zagreb, Svetošimunska Cesta 25, 10002 Zagreb, Croatia; zjanjecic@agr.hr; 3 Department of Animal Science, Biotechnical Faculty, University of Ljubljana, Groblje 3, SI-1230 Domzale, Slovenia; silvo.zgur@bf.uni-lj.si (S.Ž.); klemen.potocnik@bf.uni-lj.si (K.P.)

**Keywords:** transglutaminase, sensory analysis, meat quality

## Abstract

**Simple Summary:**

Transglutaminases are enzymes used for joining cuts or fragments of meat together to make larger pieces that are easier to handle or a product that is more attractive to consumers. They react differently with various meats and at different inclusion levels, so this study investigated quality traits of intact chicken meat and restructured chicken meat supplemented with different proportions of transglutaminase. The results showed that enzyme-supplemented restructured meat had lower cooking loss and greater tenderness compared to intact meat. Sensory attributes were not affected by the supplemented enzyme, and there was no difference in these attributes compared to intact meat. Therefore, supplementation with transglutaminase could be undoubtedly considered as a valuable contributing agent in improving yield and texture of minced meat, and reducing other additives usually used in chicken meat processing.

**Abstract:**

Transglutaminases (TG) are enzymes that improve the functional properties of proteins in meat products, contribute to the strong cohesion of meat without the further need for the addition of sodium chloride or phosphates, and have a positive effect on the texture of the meat product. This study aimed to investigate the physicochemical and sensory attributes of intact and restructured chicken meat supplemented with different TG proportions. The study was conducted on chicken breast meat samples (*n* = 40) originating from the line Ross 308. The intact samples were separated from the *pectoralis major* muscle, whereas the rest of the breast meat was ground, divided into equal parts, and supplemented with TG (0.2%; 0.4%; 0.6%; 0.8%; 1%). The intact meat had the highest cooking loss (19.84) when compared to 0.2% (15.51), 0.4% (15.04), 0.6% (14.95), 0.8% (14.95), and 1% (15.79) TG-supplemented meat. The intact meat had greater shear force (16.90) than 0.2% (5.16), 0.4% (5.39), 0.6% (5.16), 0.8% (5.98), and 1% (6.92) TG supplemented meat. There was no difference between intact meat and TG-supplemented meat in color, taste, odor, texture, and overall acceptability (*p* > 0.05). Therefore, TG supplementation can be used in improving yield and texture of minced chicken meat.

## 1. Introduction

Today’s meat industry is faced with a challenge involving modification of different processing techniques (use of improved raw materials, reformulation of products, changing the technological process) that lead to meat quality required by consumers [1,2]. In general, it is known that consumers demand healthier meat products. Currently, the emphasis is on the minimum use of additives (such as phosphates or sodium chloride) traditionally involved in the meat industry [2,3,4]. However, numerous studies reported that exclusion of sodium chloride and phosphate led to meat products with poor physicochemical properties [5,6,7]. Marques et al. [7] reported that addition of transglutaminase enzyme (TG) had been used for inducing gelation and reducing or eliminating the need to add sodium chloride and phosphates in products. Stangierski et al. [8] reported that in the case of proteins, which do not form gels with desirable rheological properties after thermal processing, their functionality might also be improved by using TG. TG catalyzes the bonding of acyl transfer reactions between the *γ*-carboxyamide group of peptides of bound glutamine residues and a variety of primary amines. The reaction results in the formation of high molecular weight polymers. In the presence of primary amines, TG can crosslink the amines to the glutamines of a protein. In the absence of primary amines, water will react as a nucleophile and lead to deamidation of glutamines. The aforementioned reactions can influence the functional properties of proteins in food [9,10,11]. It has been proven that TG improves the functional properties of proteins in meat products, contributes to the strong cohesion of a block of meat without the further need for the addition of sodium chloride or phosphates, and increasing hardness has a positive effect on the texture of the meat product [11,12,13]. Literature reports revealed that TG reacted differently with various protein sources and at different inclusion levels [14]. Furthermore, the most important findings obtained from the studies regarding TG and chicken meat involve the use of TG in combination with other additives during processing of different meat products. Lantto et al. [5] investigated the effects of laccase and TG on the firmness and weight loss of cooked chicken breast meat homogenate gels. In addition to these enzymes, meat homogenate samples were mixed with phosphate (0.3%), ascorbic acid (0.06%), glucose (0.1%), and nitrite (0.012%). Uran and Yilmaz [11] investigated the effect of TG (0.2%, 0.4%, 0.6%, 0.8%, and 1%) on the quality characteristics of chicken burgers. The chicken burgers were processed following the common procedures for burger production (grounding of fresh chicken breast meat, mixing, adding of ice, emulsion fat, burger mix, antimicrobial substance, carmine, nitrite, and filling). Tseng et al. [15] researched the effect of TG (0%, 0.05%, 0.1%, 0.2%, 0.4%, and 1.0%) on the quality of low-salt chicken meatballs. Each formulation also contained 1.0% salt, 0.2% sodium tripolyphosphate, 0.8% monosodium glutamate, 3% sugar, 0.2% white pepper, and 0.1% seasoning powder. Simultaneous application of TG (0.3%) and high pressure on chicken batters with the addition of fresh egg yolk (10%), dehydrated egg white (10%), cold water (30%), and salt (1%) was investigated by Trespalacios and Pla [16]. Uran et al. [17] investigated the effect of TG (0%, 0.5%, and 1%) on the quality properties of chicken breast patties produced with the addition of non-specified amounts of salt and different spices.

The aim of this study was to investigate the physicochemical and sensory attributes of intact chicken breast meat and restructured chicken breast meat supplemented with different proportions of TG enzyme without any other additives.

## 2. Materials and Methods

### 2.1. Raw Material and Preparation of Restructured Chicken Breast Meat

The study was conducted on chicken breast meat samples originating from 40 chicken broilers from the line Ross 308. The study was conducted in accordance with Croatian legislation (The Animal Protection Act, The Official Gazette 102/17; The Regulation on the Protection of Animals Used for Scientific Purposes, The Official Gazette 55/13), and was approved by the Bioethical Committee for the Protection and Welfare of Animals at the University of Zagreb, Faculty of Agriculture, Croatia (Class: 114-04/20-03/10; Ref. 251-71-29-02/19-20-2, 30-11-2020). The animals were slaughtered at 35 d of age. The carcasses were eviscerated and chilled in a cold chamber at 4 °C for 24 h before dissection. Chicken breast meat was manually trimmed of skin, visible fat, and connective tissue. The intact meat samples were separated from the left lateral side of the *pectoralis major* muscle and were not supplemented with TG. The samples were weighed and stored at 4 °C for 24 h in a refrigerator pending further analysis. The rest of the breast meat was coarsely ground through a plate (Ø 10 mm). Immediately after that, the ground meat was divided into five portions of equal mass and supplemented with microbial TG (Special Ingredients Ltd., Chesterfield, UK) in concentrations as follows: 0.2%, 0.4%, 0.6%, 0.8%, and 1.0%. According to the producer, this TG product is also called ‘Meat glue’ and is comprised of sodium casein (E469), maltodextrin, transglutaminase, and sunflower lecithin (E322). Each portion of ground meat was manually homogenized for 10 min to allow even distribution of TG. There were no other additives in the mixtures. During the entire processing period, temperature of the meat was controlled and did not exceed 10 °C. Each mixture of restructured chicken breast meat (RCM) was formed firmly into 10 cylindrical shapes (50 mm in diameter and 160 mm in length) without visually entrapped air using polyvinyl chloride film (PVC). The ends of the PVC film were firmly twisted. The RCMs were stored for cold binding at 4 °C for 24 h in a refrigerator. Immediately after the binding stage, the RCMs were used for further analyses.

### 2.2. Physicochemical Analyses

2.2.1. pH Value

The pH value of the samples was measured in triplicate using a penetrating electrode (InLab Solids Pro) adapted to the portable pH meter Seven2Go (Mettler Toledo, Greinfensee, Switzerland). For the pH determination, the electrode was inserted in the center of each sample.

#### 2.2.2. Color

The color parameters (L * a * b *) were successively measured in triplicate on the cross-section of the samples after a 1 h blooming period using a Chroma Meter (Minolta CR 400, Osaka, Japan), with measurements standardized with respect to the white calibration plate.

#### 2.2.3. Cooking Loss

Cooking loss (CL) was determined using a method described by Honikel [18]. Each sample was weighed, placed in a polyethylene bag, and cooked in a water bath (85 °C) until the endpoint temperature of 75 °C in the sample center was attained. After that, the samples were cooled in ice slurry, dried, and weighed. CL was calculated on the basis of the weight loss (%). The cooked samples were stored at 4 °C for 24 h in a refrigerator and used for Warner –Bratzler shear force (WBSF) determination.

#### 2.2.4. Warner–Bratzler Shear Force

The WBSF was evaluated by using an Instron Universal Testing System (Model 3345, Instron, Canton, MA, USA) equipped with a WBSF device. Each sample was cut into ten square cores (10 × 10 × 25 mm) that were sheared once perpendicular by using the Instron unit calibrated to a full scale with a 500 Newton load cell, a crosshead speed of 250 mm/min, and a sample rate of 10 points/s. The mean value of the ten replicates was taken as the maximum shear force value.

### 2.3. Sensory Evaluation

After cooking the meat samples as described above for CL, samples were evaluated by six panelists. The panel comprised students and staff of the Departments of Animal Science and Technology. The analyses were performed in a well-lit room, at a temperature of about 23 °C, and relative humidity of 60–70%. Panelists were asked to evaluate taste, color, odor, texture, and overall acceptability on a 7-point hedonic scale. The scale was defined as 1—very poor, 2—poor, 3—slightly poor, 4—fair, 5—moderate, 6—good, and 7—excellent [15]. Each sample was prepared in a uniform manner by removing the casing and cutting the cylinder into equal cross-sections (15 mm width). The samples were individually coded with three-digit numbers, and randomly presented to the panelists on a white porcelain plate. Each sample was evaluated in triplicate, i.e., three sessions. During each session, panelists were provided with water and bread to rinse and eat between tasting samples. The values were statistically calculated as median values and included in the further statistical analysis.

### 2.4. Statistical Analysis

Statistical analysis was performed using the GLM procedure of the SAS/STAT software package version 9.4 [19]. Post hoc comparison among the least square means was performed using a Bonferroni multiple test correction. The difference between means was considered significant at *p* < 0.05.

## 3. Results and Discussion

### 3.1. Physicochemical Attributes

The pH value in RCM groups supplemented with TG enzyme was significantly greater than the pH value of intact meat (Table 1). Despite this difference, it should be noted that all determined pH values in the present study are within the ‘normal’ range of chicken meat [20]. Previous research also observed that a slight increase in the pH value of the samples was accompanied by a greater dosage of the TG enzyme [16,17,21]. Setiadi et al. [21] reported that greater pH values, as a result of addition of a TG supplementation, are due to the crosslinking reaction to the sample protein, which chemically produces the ammonia base molecule, and thus the more alkaline ammonia content is able to influence the pH value. However, this trend was not observed in the studies of Trespalacios and Pla [16], Uran et al. [17], and Uran and Yilmaz [11].

Comparison of the colorimetric values showed a significant difference in lightness (L *) between intact meat and RCM groups supplemented with TG enzyme (Table 1). The intact meat had the lowest L * value (56.44). Comparing the RCM groups supplemented with TG enzyme, no change was found between 0.2% and 0.4% groups, between 0.4%, 0.8%, and 1% groups, nor between 0.6%, 0.8%, and 1% groups. Uran et al. [17] did not find statistically significant differences in the L * values of chicken patties of the non-supplemented group (41.81) and those supplemented with TG of 0.5% (43.10) and 1% (42.15). Uran and Yilmaz [11] also did not report statistically significant differences in the L * values of chicken burgers supplemented with TG of 0.2% (54.93), 0.4% (56.53), 0.6% (53.78), 0.8% (54.04), and 1% (54.64). However, they found significant difference in the L * value of the non-supplemented group when compared with those of TG supplemented groups. The L * value of the non-supplemented group was 50.29, whereas the highest L * value of chicken burgers supplemented with 0.4% of TG enzyme was 56.53.

When comparing values for redness (a *), the lowest value was found in the intact meat, which significantly differed from the 0.4% and 1.0% TG supplemented groups. Differences among all other groups were not statically significant. In chicken patties, Uran et al. [17] did not find significant difference between the a * values of the non-supplemented (7.42) group and other TG supplemented groups (0.5% = 6.98, and 1% = 6.96). In contrast, in chicken burgers Uran and Yilmaz [11] found the highest a * value for the 0.6% TG supplemented group (23.51), while the lowest value was found for the 0.4% (20.79) TG supplemented group. The a * value in chicken burgers with 0.4% TG was significantly different from the other TG supplemented groups (0.2%, 0.6%, 0.8%, and 1%). The authors pointed out that differences in a * values between the other TG supplemented groups (0.2%, 0.6%, 0.8%, and 1%) were not found (*p* > 0.05).

The results of the present study indicate that there was no significant difference between yellowness (b *) of intact meat and RCM groups supplemented with TG enzyme (*p* > 0.05; Table 1). Uran and Yilmaz [11] found the largest statistical difference in the b * value of chicken burgers in the 0.4% (9.36) and 0.8% (8.96) TG supplemented groups. Furthermore, they found statistically similar values for b * values between the non-supplemented group, and the 0.2%, 0.4%, 0.8%, and 1% TG supplemented groups, as well as the 0.6% and 0.8% TG supplemented groups. Uran et al. [17] did not find statistically significant difference in the b * values of chicken patties of the non-supplemented group and the 0.5% TG supplemented group. When the TG supplemented groups were evaluated, there was significant difference in b * values between the 0.5% (24.42) and 1% (23.07) TG supplemented groups.

Regarding CL in the present study, there was no significant difference in water-holding capacity between RCM groups supplemented with different TG enzyme % (Table 1). Interesting, the results revealed that the intact meat had the highest CL value (19.84) when compared to 0.2% (15.51), 0.4% (15.04), 0.6% (14.95), 0.8% (14.95), and 1% (15.79) TG supplemented RCMs. This result indicates that the ground meat supplemented with different TG could certainly have greater yield than the whole meat piece. It is valuable information that could be considered in processing of different types of ground (restructured) meat products (e.g., meatballs, patties, hamburgers, different types of sausages) for gaining greater yields without/with minimum use of other additives, such as phosphates, sodium chloride, or monosodium glutamate. Pietrasik et al. [22] and Mostafa [23] reported that TG enzyme increased the water-holding capacity of meat products by decreasing cooking and thawing losses. In the case of raw materials with the addition of TG, the gel-forming capability was improved and thus, indirectly, the water-holding capacity was improved as well. These authors pointed out that by improving ε- (γ-glutamyl) lysine peptide bonds, more water is retained, despite the temperature at which processing was performed. In accordance with this, Uran and Yilmaz [11] in chicken burgers, and Uran et al. [17] in chicken patties also confirmed that TG supplementation significantly decreased CL in comparison to the non-supplemented ground meat. Stangierski et al. [8], Stangierski and Baranowska [24], Stangierski et al. [25], and Stangierski and Kaczmarek [26] indicated that pre-incubation time, the amount of supplemented TG, and heating treatment are also major factors that could influence cooking loss and texture properties of the meat. Stangierski and Kaczmarek [26] investigated the effect of TG (0.1%, 0.2%, 0.3%, and 0.6%) on the quality of poultry surimi during an incubation time of 1, 3, 5, 8, and 24 h, and an incubation temperature of 6–7 °C. They found that a 0.3% concentration of TG was the most advantageous in reducing cooking loss from poultry surimi gels. Stangierski et al. [8] found that poultry meat supplemented with 0.3% of TG, pre-incubated for 3 h, and thermally processed at 70 °C had low cooking loss and improved texture properties.

The results of our study indicated that a slight increase in the WBSF of the RCM samples was accompanied by a greater dosage of the TG enzyme (Table 1). When statistically evaluated, there was no significant difference in WBSF among RCMs supplemented with TG enzyme. Given that one of the key properties of TG, and also the reason for its use in the meat industry, is the binding of myofibrils, increasing gel structure and thus increasing texture [8,22,25], a greater WBSF value in RCM with a higher proportion of TG was actually expected. The results also showed that intact meat had a significantly greater WBSF than the RCMs supplemented with TG enzyme. With regard to this, it is interesting that the RCMs with the highest enzyme dosage (1%) had significantly lower WBSF values than intact meat. Tseng et al. [15] found that the gel strength of low-salt chicken meatballs increased with increasing TG enzyme supplementation (0.05%, 0.1%, 0.2%, 0.4%, and 1%), and at proportions above 0.2% was significantly higher than the non-supplemented group. Uran and Yilmaz [11] found no statistically significant difference in WBSF between the non-supplemented group and with 0.2% and 0.4% TG enzyme groups, nor between 0.6% and 0.8% TG enzyme groups. They found the largest statistically significant difference in the WBSF of chicken burgers supplemented with 1% TG enzyme. Uran et al. [17] also found statistically significant difference in the WBSF of chicken patties supplemented with 0.5% and 1% TG enzyme, respectively. Significantly higher WBSF was found in chicken patties supplemented with 1% TG enzyme. Compared to non-supplemented group, chicken patties supplemented with 1% TG enzyme had greater WBSF values. These greater differences between researchers could be due to the fact that, except with TG, the samples were prepared with different additive supplementation (emulsion fat, carmine, nitrite, salt, monosodium glutamate, sodium tripolyphosphate, etc.). Furthermore, these differences in WBSF values could be related to the fact that in the present study the intact meat samples were used as the samples without TG supplementation, while in the each of the above-mentioned studies the non-supplemented group was mechanically treated in the same manner as the supplemented groups of the relevant studies. Surely, as reported by Stangierski et al. [8], Stangierski and Baranowska [24], and Stangierski et al. [25], differences in WBSF could be the result of other aforementioned factors that were found to have an effect on textural properties on the meat supplemented with TG but were not considered in the present study nor in the other aforementioned comparable studies.

### 3.2. Sensory Evaluation

According to the results, there was no significant difference between the intact meat and the RCM groups supplemented with TG enzyme in terms of color, taste, odor, texture, and overall acceptability (*p* > 0.05). Except for the color, the mean values between treatments ranged from ‘moderate’ to ‘good’ (5 = moderate; 6 = good; Table 2.). The results of the present study revealed that the increase in the proportion of TG did not cause any negative acceptance in terms of evaluated sensory attributes. Uran and Yilmaz [11] confirmed that TG in different proportions (0%, 0.2%, 0.6%, 0.8%, and 1%) did not significantly affect the color, taste, odor, texture, and general evaluation of chicken burgers. They also indicated that TG in chicken burgers did not cause any negative acceptance in evaluated sensory attributes (the same hedonic scale as in the present study). Tseng et al. [15] investigated the effect of TG supplementation (0%, 0.05%, 0.1%, 0.2%, 0.4%, and 1%) on the sensory properties of chicken meatballs. It was found that the addition of TG in different proportions did not significantly affect the appearance, color, and taste of chicken meatballs (*p* > 0.05). However, the results of that study showed that the meatballs supplemented with 1% of TG significantly differed from the other samples in texture, juiciness, and overall acceptability. These findings indicated that chicken meatballs with 1% TG had the highest gel strength, more complete gel clusters, and the highest yield. It was also noticed that there were no negative scores for the evaluated attributes (the same hedonic scale as in the present study) and they were predominantly scored as ‘good’.

## 4. Conclusions

The present study revealed varying changes in pH value, color parameters (except for b * value), cooking loss, and shear force of intact meat and RCMs supplemented with different proportions of TG enzyme. The most interesting change was related to lower cooking loss and shear force values of RCMs supplemented with different proportions of TG enzyme compared to intact meat. Since there was no significant change in cooking loss and shear force values among RCM groups supplemented with different proportions of TG enzyme, lower inclusion rates could be considered in different processing technology. Sensory attributes were not affected by the supplemented enzyme, and there was no difference in these attributes compared to intact meat. Therefore, TG enzyme supplementation could undoubtedly be considered as a valuable contribution agent in improving yield and texture of minced meat, and reducing other additives usually used in chicken meat processing. However, taking into consideration the changes in physicochemical attributes, it is important to consider further investigations that could give precise information about variations in meat quality attributes which are affected by the inclusion of TG enzyme.

## Figures and Tables

**Table 1:** pH value (pH), color parameters (L * a * b *), cooking loss (CL), and Warner–Bratzler shear force (WBSF) of intact chicken breast meat and restructured chicken breast meat (RCM) supplemented with 0.2%, 0.4%, 0.6%, 0.8%, and 1% of transglutaminase enzyme (LSM ± SE).

	Treatments					
	0	0.2	0.4	0.6	0.8	1.0
**Attribute**						
pH	5.82 ± 0.01 ^a^	5.88 ± 0.01 ^b^	5.89 ± 0.01 ^b^	5.91 ± 0.01 ^b^	5.90 ± 0.01 ^b,^	5.87 ± 0.01 ^b^
L *	56.44 ± 0.35 ^a^	63.53 ± 0.35 ^b,d^	63.39 ± 0.35 ^b,e^	61.83 ± 0.35^c^	62.02 ± 0.35 ^c,de^	61.97 ± 0.35 ^c,e^
a *	11.53 ± 0.34 ^a^	12.33 ± 0.34 ^a,b^	13.00 ± 0.34 ^b,c^	12.38 ± 0.34 ^a,c^	12.33 ± 0.34 ^a,b^	12.79 ± 0.34 ^b,c^
b *	20.20 ± 0.51	19.62 ± 0.51	20.44 ± 0.51	19.50 ± 0.51	19.06 ± 0.51	19.69 ± 0.51
CL, %	19.84 ± 0.90 ^a^	15.51 ± 0.90 ^b^	15.04 ± 0.90 ^b^	14.95 ± 0.90 ^b^	15.97 ± 0.90 ^b^	15.79 ± 0.90 ^b^
WBSF, N	16.90 ± 0.41 ^a^	5.16 ± 0.41 ^b^	5.39 ± 0.41 ^b^	5.16 ± 0.41 ^b^	5.98 ± 0.41 ^b^	6.92 ± 0.41 ^b^

^a, b, c, d, e^ LSM with different letters in the same row significantly differ (Bonferroni post hoc test, *p* < 0.05); *n* = 10.

**Table 2:** Sensory attributes of intact chicken breast meat and restructured chicken breast meat (RCM) supplemented with 0.2%, 0.4%, 0.6%, 0.8%, and 1% of transglutaminase enzyme (LSM ± SE) ^†^.

	Treatments					
	0	0.2	0.4	0.6	0.8	1.0
**Attribute**						
Color	6.57 ± 0.66	5.28 ± 0.66	5.71 ± 0.66	4.92 ± 0.66	5.00 ± 0.66	5.85 ± 0.66
Taste	6.14 ± 0.63	5.57 ± 0.63	5.00 ± 0.63	5.28 ± 0.63	5.28 ± 0.63	5.57 ± 0.63
Odor	6.00 ± 0.55	6.42 ± 0.55	6.28 ± 0.55	5.57 ± 0.55	5.57 ± 0.55	5.71 ± 0.55
Texture	6.00 ± 0.58	5.42 ± 0.58	4.85 ± 0.58	5.42 ± 0.58	5.71 ± 0.58	5.57 ± 0.58
Overall Acceptability	6.14 ± 0.51	5.14 ± 0.51	5.00 ± 0.51	5.21 ± 0.51	5.28 ± 0.51	5.35 ± 0.51

^†^ There were no statistical differences between the presented least square means (*p* > 0.05); *n* = 10.

## Data Availability

The data that support the findings of this study are available from the corresponding author, A.K., upon reasonable request.
